# Micron-Scale Anomalous Hall Sensors Based on Fe*_x_*Pt_1−*x*_ Thin Films with a Large Hall Angle and near the Spin-Reorientation Transition

**DOI:** 10.3390/nano11040854

**Published:** 2021-03-27

**Authors:** Kang Wang, Yiou Zhang, Shiyu Zhou, Gang Xiao

**Affiliations:** Department of Physics, Brown University, Providence, RI 02912, USA; kang_wang@brown.edu (K.W.); yiou_zhang@brown.edu (Y.Z.); shiyu_zhou@brown.edu (S.Z.)

**Keywords:** anomalous Hall sensors, FePt alloys, perpendicular magnetic anisotropy, spin–orbit coupling, noise characterizations, micro-sensing and imaging

## Abstract

In this work, we fabricate and characterize an energy-efficient anomalous Hall sensor based on soft-magnetic Fe*_x_*Pt_1−*x*_ thin films with a large anomalous Hall angle. By varying the composition of the Fe*_x_*Pt_1−*x*_ alloy, its layer thickness and interfacial materials, the magnetization is tuned to be near the spin transition between the perpendicular and in-plane reorientations. We performed magneto-transport and noise characterizations on anomalous Hall sensors with a small sensing area of 20 × 20 µm^2^ in the 180 to 350 K temperature range. We found the best performance in a 1.25-nm-thick Fe_0.48_Pt_0.52_ sandwiched by two 1.6-nm-thick MgO layers at room temperature. The sensor has a large anomalous Hall angle of 1.95%. Moreover, it has the best field detectability of 237.5 nT/√Hz at 1 Hz and 15.3 nT/√Hz at 10 kHz, as well as a high dynamic reserve of 112.0 dB. These results suggest that the Fe*_x_*Pt_1−*x*_ alloy system is suitable for energy-efficient anomalous Hall sensors, particularly in micro-sensing applications.

## 1. Introduction

Anomalous Hall sensors are magnetic sensors that take advantage of the anomalous Hall effect of ferromagnets [1,2,3,4,5,6]. Electrons with different spins, under the spin–orbit interaction, deflect towards opposite transverse directions when a longitudinal electric current I is applied [7]. This gives rise to an anomalous Hall voltage ($V_{H}=R_{H}I$, where $R_{H}$ is the Hall resistance), which is proportional to the perpendicular magnetization that can be controlled by the external perpendicular magnetic field. For a given magnetic system, the Hall-resistance response, also known as the sensitivity *s* (in the unit of Ω/T), depends strongly on the magnetic anisotropy of the ferromagnet [1,2,3,4,5,6]. The sensitivity, together with the Hall voltage noise $S_{V}$ (in the unit of V^2^/Hz), including both the electronic noise originating from defects and the magnetic noise originating from thermal magnetic fluctuations, determines the magnetic field detectability $S_{T}^{0.5}=S_{V}^{0.5}/Is$ [2,4,5].

In recent years, anomalous Hall sensors have been studied in multiple magnetic systems with different magnetic anisotropies [1,2,3,4,5,6]. In the in-plane magnetized materials, although the sensitivity is low compared to conventional semiconductor Hall sensors, the voltage noise is low as well. As a result, the field detectability is reasonably good at low frequencies [4]. On the other hand, the sensitivity increases with the emergence of the perpendicular magnetic anisotropy (PMA), which has the tendency to increase the voltage noise as well [2,5]. The competition between the two can, in fact, reduce the field detectability. In our recent studies on Ta/Co_40_Fe_40_B_20_/MgO [5] and MgO/Co_40_Fe_40_B_20_/Ta/Co_40_Fe_40_B_20_/MgO [2] systems, we found that anomalous Hall sensors have the best performance when the magnetization is tuned to be near the spin-reorientation transition. In Ta/Co_40_Fe_40_B_20_/MgO [5], the field detectability reaches 76 nT/√Hz at 1 Hz and 2 nT/√Hz at 10 kHz at a perpendicular bias field of −12 Oe. In MgO/Co_40_Fe_40_B_20_/Ta/Co_40_Fe_40_B_20_/MgO, where a weak ferromagnetic interlayer exchange coupling (IEC) exists between two ferromagnetic layers [2], the field detectability reaches 126.1 nT/√Hz at 1 Hz and 4.5 nT/√Hz at 1 kHz at zero bias field. Moreover, the interlayer exchange-coupled magnetic thin films have a small temperature coefficient of sensitivity of 530 ppm/K, as well as a low cross-field error [2].

The field detectability of anomalous Hall sensors is not as good as magneto-resistive (MR) sensors with large sensing areas [2,8,9,10,11]. However, normalized by sensing areas, anomalous Hall sensors perform comparably or better over MR sensors in micro-sensing applications where a high spatial resolution is desired. Furthermore, anomalous Hall sensors are sensitive to the perpendicular magnetic field. This is a complementary to MR sensors, which are sensitive to the in-plane field. The integration of these two types of sensors offers an avenue to the implementation of three-axis magnetic vector sensing.

Although anomalous Hall sensors studied so far have a good sensing performance, the anomalous Hall angle, which is defined as the ratio of the Hall resistivity to the longitudinal resistivity, has been low. The anomalous Hall angle of the Co_40_Fe_40_B_20_ system is only 0.7% [2]. Assuming the same magnetization response to the perpendicular magnetic field and considering the same voltage Johnson noise (independent on the current) at high frequencies, fabricating anomalous Hall sensors based on ferromagnets with large anomalous Hall angles can increase the field sensitivity, thus reducing the need for a large longitudinal current to achieve the same field sensing detectability. A large Hall angle makes the magnetic sensing energy efficient.

The anomalous Hall signal originates either from the gauge field arising from the electronic band (the intrinsic mechanism) [12,13,14,15,16,17,18] or from the electron scattering due to the spin–orbit interaction at impurities (the extrinsic mechanism) [19,20,21,22,23,24,25,26,27]. In the intrinsic regime, the Berry curvature sets a limitation to the Hall voltage response, and a large Hall signal was only observed in materials such as magnetic topological insulators at extremely low temperatures [17,18]. In the extrinsic regime, the anomalous Hall angle depends on the spin–orbit interaction strength of ferromagnets. The anomalous Hall angle is typically small (0.1~1%) because of the small spin–orbit interaction strength [2,19,20,22,23,24]. It has been found that by doping Pt with a large spin–orbit interaction in Fe, the anomalous Hall angle can be enhanced, while the magnetization will not suffer a large reduction as shown in our previous studies [25,26,27]. This is because Pt nearly satisfies the Stoner criterion and thus can be easily spin polarized by adjacent Fe atoms [28]. A large anomalous Hall angle of about 5% has been reported in a 30 nm thick soft-magnetic Fe_29_Pt_71_ at room temperature [25]. To lower the demagnetization energy, the Fe_29_Pt_71_ thin film is in-plane magnetized. The Fe_29_Pt_71_ thin film-based anomalous Hall sensor has a field detectability of 7 µT/√Hz at 1 Hz and 50 nT/√Hz at 1 kHz, and outperforms conventional semiconductor Hall sensors in the 31 to 500 Hz frequency range [4]. Interface engineering by neighboring the magnetic layer with oxide layers can promote PMA in the magnetic layer [29,30,31,32,33], due to the interfacial electronic hybridization and spin–orbit interaction. It has been shown that sandwiching FePt alloys with SiO_2_ layers can introduce PMA [1]. The maximum sensitivity reaches 12,000 Ω/T in SiO_2_/Fe_0.425_Pt_0.575_(1.8 nm)/SiO_2_ after 320 °C annealing [1]. However, noise in the soft-magnetic FePt alloys with the enhanced PMA has not yet been studied.

In this work, we investigated anomalous Hall sensors based on soft-magnetic Fe*_x_*Pt_1−*x*_ alloys over the whole composition range (0 < *x* < 1), with a variable layer thickness, and interfaced with different oxide layers (MgO and SiO_2_). We characterized their magneto-transport and noise properties in the 180 to 350 K temperature range that encompasses a spin-reorientation transition between perpendicular and in-plane configurations. We found that the Fe*_x_*Pt_1−*x*_-based anomalous Hall sensors are energy efficient while remaining a good sensing performance in micro-sensing applications.

## 2. Materials and Methods

To fabricate Fe*_x_*Pt_1−*x*_-based anomalous Hall sensors, we deposited multilayers in the forms of MgO(1.6)/Fe*_x_*Pt_1−*x*_(*t*_FePt_)/MgO(1.6)/TaO*_x_*(1.0), SiO_2_(5.0)/Fe*_x_*Pt_1−*x*_(*t*_FePt_)/SiO_2_(10.0)/TaO*_x_*(1.0), and Ta(1.6)/Fe*_x_*Pt_1−*x*_(*t*_FePt_)/MgO(1.6)/TaO*_x_*(1.0), respectively, on thermally oxidized silicon wafers using a home-made high-vacuum magnetron sputtering system. Numbers in brackets are layer thicknesses in nanometers. The base vacuum pressure was around $1\times 10^{-7}$ Torr. Fe*_x_*Pt_1−*x*_ layers were deposited using a target composed of several sectors of Fe and the remaining sectors of Pt. Fe and Pt were sputtered simultaneously from surfaces of corresponding sectors onto oxidized silicon wafers. Compositions of Fe*_x_*Pt_1−*x*_ were controlled by varying relative surface areas of Fe and Pt sectors. The actual composition of an Fe*_x_*Pt_1−*x*_ film was confirmed through the energy-dispersive X-ray spectroscopy (EDS, Quattro ESEM (Thermo Fisher Scientific, Waltham, MA, USA)) measurement. The deposition rate was calibrated by using the X-ray reflectivity (XRR, Bruker D8 (Bruker AXS Inc., Madison, WI, USA)) measurement. We deposited the Fe*_x_*Pt_1−*x*_ and the Ta layer using a 15- and a 3-Watt DC power (Advanced Energy, Georgetown, MA, USA), respectively, under an argon pressure of 1.4 mTorr. MgO layers were deposited directly from a MgO target using a radio-frequency (RF) power (Advanced Energy, Georgetown, MA, USA) under an argon pressure of 0.7 mTorr. SiO_2_ layers were deposited from a Si target using a RF power under a total argon and oxygen pressure of 4 mTorr. During the SiO_2_ deposition, the flow-rate ratio between argon and oxygen was 2.9:1.0 (argon: oxygen).

After deposition, we processed thin film stacks into Hall crosses with an active area of 20 × 20 µm^2^, using photolithography (MLA150 Maskless Aligner (Heidelberg Instruments, Torrance, CA, USA)) and physical ion milling (MIM-TLA20 (PVA TePla/Technics, Corona, CA, USA)). We deposited Cr/Au as electrodes in order to reduce the Johnson noise that was positively correlated with the resistance between the two Hall voltage leads [34,35,36]. Finally, these samples were thermally annealed in a home-made high-vacuum chamber at different temperatures$\;T_{a}$. A perpendicular magnetic field of 0.4 T was applied during annealing processes.

We performed both magneto-transport and noise measurements in a Quantum Design^®^ Physical Property Measurement System (Quantum Design, San Diego, CA, USA). Instead of extracting the field sensitivity from a Hall curve, we measured the sensitivity using an AC method, as described previously in detail [2,5]. This method provides an accurate measurement of the sensitivity, while the sensitivity derived from the slope of the Hall curve may be overestimated [37,38,39]. Noise measurements using a two-channel cross correlation method [40] have also been described in detail in our previous works [2,5,11]. In both measurements, we applied a 1 mA current to Hall crosses. The voltage noise ($S_{V}=I^{2}S_{R}$) at low frequencies increases with increasing current. However, assuming a constant resistance noise ($S_{R}$), the field detectability ($S_{T}^{0.5}=S_{V}^{0.5}/Is=S_{R}^{0.5}/s$) would be independent of the current. On the other hand, the voltage noise at high frequencies where the Johnson noise is dominant is independent of the current. This would give rise to an improved field detectability at high frequencies with increasing current.

## 3. Results and Discussion

**Figure 1:**
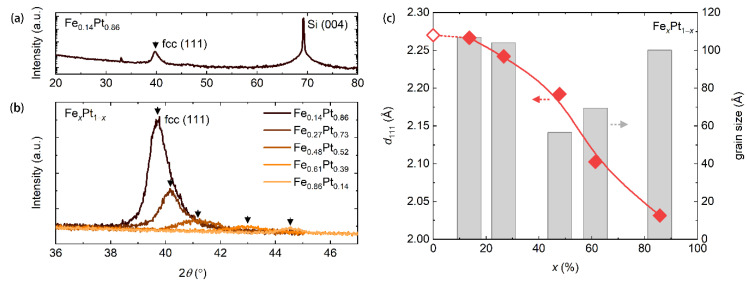
(**a**) The X-ray diffraction result for a 30 nm thick Fe_0.14_Pt_0.86_ thin film. The black arrow is used to denote the peak position of (111) reflection of the face-centered cubic Fe_0.14_Pt_0.86_ alloy. (**b**) X-ray diffraction results for Fe*_x_*Pt_1−*x*_ thin films with different compositions. Black arrows are used to denote peak positions of (111) reflection of the face-centered cubic alloys. (**c**) The (111) lattice constant (dots and the red line) and crystalline dimensions (grain sizes) of Fe*_x_*Pt_1−*x*_ thin films. The red line is used to guide eyes. The open dot is extracted from reference [41].

We first gained insight into the crystalline structures of the soft-magnetic Fe*_x_*Pt_1−*x*_ alloys with different compositions. For this study, we deposited 30 nm thick Fe*_x_*Pt_1−*x*_ films on thermally oxidized silicon wafers. Growth conditions were the same as in previous descriptions. The X-ray diffraction (XRD, Bruker D8 (Bruker AXS Inc., Madison, WI, USA)) measurements were performed for this study and the results are presented in Figure 1a,b. For Fe_0.14_Pt_0.86_, only one peak around 2θ=40° (θ is the diffraction angle) is observed in the 2θ-angle range from 20° to 80°. This peak corresponds to the (111) reflection of the face-centered cubic structure of Fe_0.14_Pt_0.86_. The peak position shifts to large angles when the Fe concentration increases, inferring a decrease in the (111) lattice constant, as presented in Figure 1c, which was derived from peak positions with the X-ray wavelength of 1.54 Å. This can be easily understood since Fe atoms have a smaller size compared to Pt atoms. In addition to the peak-position shift, the peak intensity decreases with increasing the Fe concentration. This is possibly because of different structures of Pt (face-centered cubic) and Fe (body-centered cubic) at room temperature [41,42]. We derived crystalline dimensions (grain sizes) of these alloys from the full width at half maximum of these peaks through the Scherrer equation. Crystalline dimensions of these alloys do not show a great change as varying compositions, remaining in the 60 to 100 Å range. These results suggest a polycrystalline property of the soft-magnetic Fe*_x_*Pt_1−*x*_ alloys in our studies.

**Figure 2:**
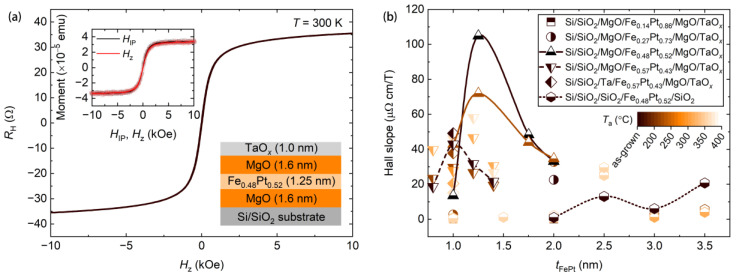
(**a**) Hall resistance $R_{H}$ vs. perpendicular magnetic field $H_{z}$ for a multilayer stack of MgO(1.6)/Fe_0.48_Pt_0.52_(1.25)/MgO(1.6)/TaO*_x_*(1.0). The result was measured at room temperature. The right insert shows the schematic of the multilayer structure. The left insert shows magnetization curves as a function of the in-plane magnetic field $H_{\mathrm{IP}}$ (black) and the perpendicular magnetic field$\;H_{z}$ (red). (**b**) Hall slopes at zero magnetic field for different multilayers, obtained from Hall curves. Different symbols represent results for different multilayers and different colors represent results for samples that were annealed at different temperatures. Lines are used to guide eyes.

Magnetically, a single Fe*_x_*Pt_1−*x*_ layer would have an in-plane magnetic anisotropy to lower the demagnetization energy [4,25,26,27]. Theoretical studies suggest that neighboring the magnetic layer with oxide layers can promote the PMA [30], due to electronic hybridization between *d* electrons of the magnetic layer and 2*p* electrons of the oxygen, as well as to the spin–orbit interaction at the interface. The improved PMA in the soft-magnetic Fe*_x_*Pt_1−*x*_ alloys has been experimentally observed in SiO_2_/Fe*_x_*Pt_1−*x*_/SiO_2_ [1]. The PMA in the magnetic multilayer is further enhanced by thermal annealing processes [1]. In our studies, we fabricated Fe*_x_*Pt_1−*x*_ thin films with the Fe*_x_*Pt_1−*x*_ layer sandwiched either by two MgO layers, by two SiO_2_ layers, or by one MgO layer and one Ta layer, as described in the experimental section. We measured Hall resistance $R_{H}$ as a function of the perpendicular magnetic field $H_{z}$ through Hall crosses. Figure 2a shows a Hall curve for a typical multilayer stack of MgO(1.6)/Fe_0.48_Pt_0.52_(1.25)/MgO(1.6)/TaO*_x_*(1.0). For this multilayer structure, the saturated Hall resistance reaches 35.51 Ω, corresponding to a Hall resistivity of 7.99 µΩ cm and a large Hall angle of 1.95% based on the longitudinal resistivity of 408.90 µΩ cm in this sample. The Hall angle is almost three times larger than that of the Co_40_Fe_40_B_20_ system [2], suggesting that Fe*_x_*Pt_1−*x*_ alloys can be a good candidate for energy-efficient anomalous Hall sensors.

To study how magnetic anisotropies depend on the Fe*_x_*Pt_1−*x*_ composition, the layered structure and the magnetic annealing condition, we calculated Hall slopes, in the unit of µΩ cm/T, at zero field from Hall curves, as presented in Figure 2b. In general, for a given multilayer, the Hall slope increases first and then decreases with the increasing Fe*_x_*Pt_1−*x*_ layer thickness *t*_FePt_. This variation trend has also been observed in many other magnetic thin films [1,43]. The decrease in the Hall slope with increasing *t*_FePt_ can be explained by the interfacial origination of the PMA. The effective PMA coefficient is given by $K_{\mathrm{eff}}=K_{s}/t_{\mathrm{FePt}}-\frac{1}{2}\mu_{0}M_{S}^{2}$ ($K_{s}$ is the interfacial PMA, $\mu_{0}$ the vacuum permeability and $M_{S}$ the saturation magnetization), which is expected to decrease with increasing *t*_FePt_. On the other hand, the decrease in the Hall slope with decreasing *t*_FePt_ in the low *t*_FePt_ range can be understood from the possible transition to the paramagnetic or superparamagnetic phase, as observed in other magnetic thin films [11,43]. The largest Hall slope among all these samples reaches 104.9 µΩ cm/T, in an as-grown multilayer of MgO(1.6)/Fe_0.48_Pt_0.52_(1.25)/MgO(1.6)/TaO*_x_*(1.0). This value is larger than that of the in-plane magnetized Fe*_x_*Pt_1−*x*_ (with a layer thickness of 30 nm) by one order of magnitude [4,25,26,27]. This confirms the enhanced PMA by neighboring Fe*_x_*Pt_1−*x*_ with oxide layers. Here we note that, in the text, we refer to the negative effective PMA as the in-plane magnetic anisotropy. We measured both in-plane and perpendicular components of the magnetization of the multilayer through a vibrating-sample magnetometer, to gain more insight into the magnetic anisotropy. The results are presented in the insert in Figure 2a. Magnetization curves as a function of the in-plane magnetic field $H_{\mathrm{IP}}$ and as a function of the perpendicular magnetic field $H_{z}$, are nearly identical to each other. This suggests that the magnetization in the multilayer structure is close to the spin-reorientation transition with a vanishing effective PMA which can be obtained from the area between the two curves. In the following section, we investigate the magneto-transport and noise properties of the multilayer structure. Before that, we would like to point out that the Hall slope of Fe*_x_*Pt_1−*x*_ sandwiched by two SiO_2_ layers is low, possibly owing to inappropriate growth conditions for this multilayer structure. Either over-oxidization or under-oxidization at the interface between the magnetic layer and the oxide layer can lower the PMA in the magnetic layer [29].

**Figure 3:**
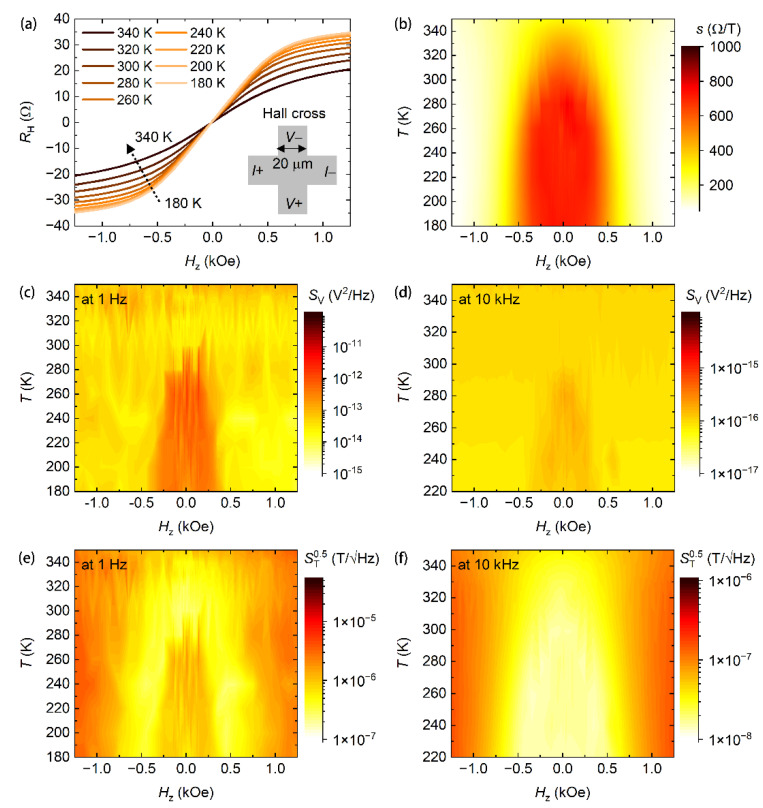
(**a**) Hall curves measured at different temperatures (180 to 340 K with a temperature interval of 20 K) for a multilayer stack of MgO(1.6)/Fe_0.48_Pt_0.52_(1.25)/MgO(1.6)/TaO*_x_*(1.0). The insert shows the schematic of the Hall cross. Maps of (**b**) the sensitivity *s*, (**c**) Hall voltage noise $S_{V}$ at 1 Hz, (**d**) Hall voltage noise $S_{V}$ at 10 kHz, (**e**) field detectability $S_{T}^{0.5}$ at 1 Hz and (**f**) field detectability $S_{T}^{0.5}$ at 10 kHz, as a function of temperature *T* and the perpendicular magnetic field $H_{z}$. In (**d**) and (**f**), only results in the temperature range from 220 to 350 K are given.

**Figure 4:**
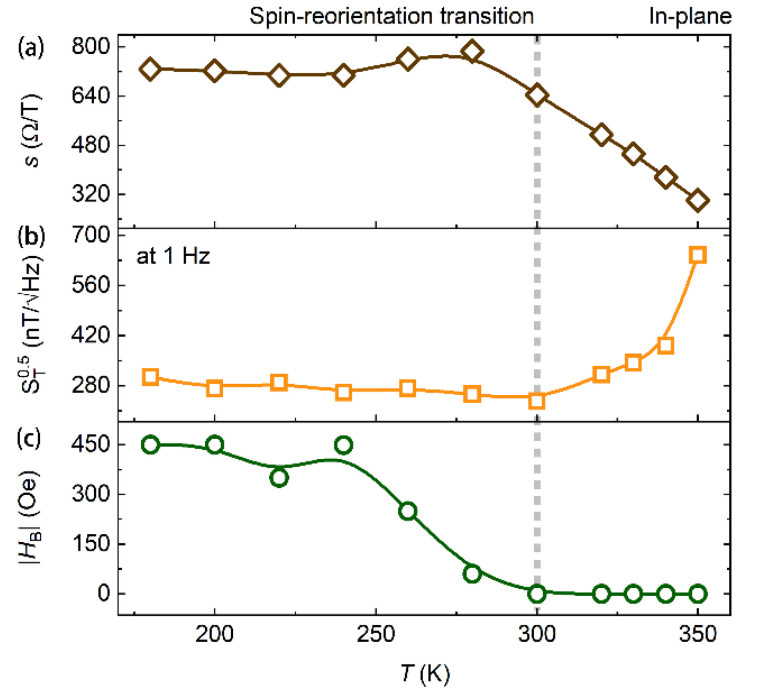
(**a**) Sensitivity *s*, (**b**) magnetic field detectability $S_{T}^{0.5}$ at 1 Hz, and (**c**) the bias field $H_{B}$ at which the best field detectability is achieved, as a function of temperature T. The vertical gray dotted line represents the temperature (300 K) where the anomalous Hall sensor attains the best performance.

We performed magneto-transport and noise measurements on the multilayer of MgO(1.6)/Fe_0.48_Pt_0.52_(1.25)/MgO(1.6)/TaO*_x_*(1.0) in the 180 to 350 K temperature range. Varying temperature can also tune magnetic anisotropies in magnetic thin films [5,31,44,45,46]. We note that the experiments in the 320 to 350 K temperature range (with a temperature interval of 10 K) and in the 180 to 300 K temperature range (with a temperature interval of 20 K) were performed separately for two Hall crosses in a single batch of the sample. Figure 3a shows Hall curves for this multilayer structure at different temperatures. The saturated Hall resistance decreases with increasing temperature. This is because the Hall resistance is dominated by the anomalous Hall resistance that is proportional to the perpendicular magnetization [7], and the saturated magnetization decreases with increasing temperature [25]. Moreover, the saturation field increases with increasing temperature, inferring the decrease in the PMA. We measured the sensitivity of the sensor at different temperatures and at different magnetic fields. The result is presented in Figure 3b. The sensitivity at zero field increases from 301.8 to 644.1 Ω/T when the temperature is decreased from 350 to 300 K. The sensitivity then remains almost constant around 750 Ω/T in a temperature range from 280 to 180 K. The temperature-dependent sensitivity at zero field has also been plotted in Figure 4a to give a clearer presentation. The sensitivity is smaller than that of magnetic thin films with larger perpendicular magnetic anisotropies [1,2,5], but still larger than that of conventional semiconductor Hall sensors [47,48,49]. Surprisingly, the anomalous Hall sensor has a good temperature stability in sensitivity in the 180 to 280 K temperature range. The temperature coefficient of sensitivity Δs/sΔT is calculated to be about 1003 ppm/K in this temperature range. This value is comparable to that of the interlayer exchange-coupled magnetic thin films (530 ppm/K) [2]. The temperature stability is usually low in single magnetic layer systems [5]. The high temperature stability in the Fe_0.48_Pt_0.52_ system is possibly owing to neighboring both sides of the Fe_0.48_Pt_0.52_ layer with MgO layers, sharing the similar mechanism of the high temperature stability in interlayer exchange-coupled magnetic thin films [2]. We derived the dynamic range $2H_{\mathrm{DRg}}$ of the anomalous Hall sensor through the full width at half maximum of the field dependence of the sensitivity. The dynamic range reaches 949 Oe at room temperature (300 K) and increases to 1566 Oe at 350 K. Again, the dynamic range remains almost constant around 1050 Oe in the 180 to 280 K temperature range.

**Figure 5:**
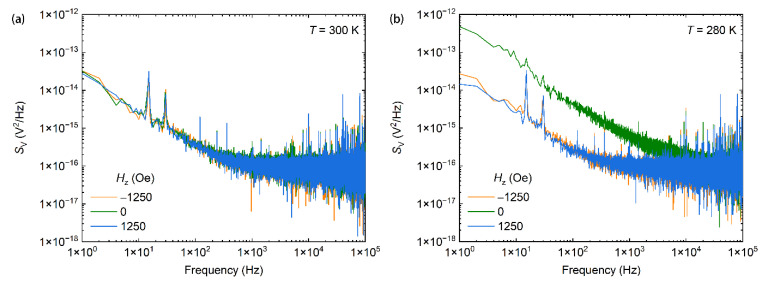
(**a**) Hall voltage noise spectra measured at 300 K and at different perpendicular magnetic fields. (**b**) Hall voltage noise spectra measured at 280 K and at different perpendicular magnetic fields.

We then measured the Hall voltage noise of the anomalous Hall sensor at different magnetic fields in the 180 to 350 K temperature range. Figure 5a,b show representative Hall voltage noise spectra at 300 K and 280 K, respectively. All spectra show 1/*f* noise at low frequencies and white Johnson noise at high frequencies. This spectral feature has also been observed in Hall voltage noise measurements at other temperatures in the temperature range in our studies. Field and temperature-dependent Hall voltage noises at 1 Hz and 10 kHz are summarized in Figure 3c,d, respectively. At low temperatures (below 300 K), the Hall voltage noise around the zero magnetic field is larger than that at a high magnetic field (Figure 3c,d and Figure 5b). This suggests that a large magnetic noise originating from thermal magnetic fluctuations exists in the anomalous Hall sensor. At high temperatures, the Hall voltage noise remains almost constant in all of the field range (Figure 3c,d and Figure 5a), suggesting that the magnetic noise is lower than the electronic noise from defects in the Fe_0.48_Pt_0.52_ layer or at interfaces. In Figure 3d, the Hall voltage noise at a large magnetic field increases with increasing temperature. This is a result of increased thermal fluctuations. The temperature-dependent Hall voltage noises in Figure 3c,d, together with the temperature-dependent sensitivity in Figure 3b, gives an indication that the anomalous Hall sensor has possibly the best performance around room temperature, at which the magnetization in Fe_0.48_Pt_0.52_ is near the spin-reorientation transition.

From the derived sensitivity and the Hall voltage noise, the field detectability can be obtained through $S_{T}^{0.5}=S_{V}^{0.5}/Is$, as presented in Figure 3e,f for the results at 1 Hz and 10 kHz, respectively. In all of the temperature ranges, the best field detectability reaches 237.5 nT/√Hz at 1 Hz and 15.3 nT/√Hz at 10 kHz at zero magnetic field at 300 K. Above this temperature, the field detectability value increases with increasing temperature due to the decrease in the sensitivity. Below 300 K, the best field detectability at a given temperature remains at a low value, as presented in Figure 4b; however, the best field detectability is only achieved when a finite perpendicular bias field $H_{B}$ is applied, as presented in Figure 3e,f. The temperature dependent $H_{B}$ has also been plotted in Figure 4c.$\;H_{B}$ remains at zero at high temperatures and starts increasing with decreasing temperature from 300 K. Zero bias field is desired in magnetic field sensing applications. All results above suggest that the anomalous Hall sensor has the best performance at room temperature where the magnetization is near the spin-reorientation transition. In the reference, Lu et al. reported a higher PMA in SiO_2_/Fe*_x_*Pt_1−*x*_/SiO_2_, which induces a high sensitivity up to 12,000 Ω/T [1]. However, we note that the sensitivity derived from the Hall slope is possibly overestimated (see discussions in the Materials and Methods section). Moreover, the Hall voltage noise is possibly large, as well. The competition between the two may not lead to an expected high field detectability, as inferred from this study and our previous works on Co_40_Fe_40_B_20_ [2,5].

Notably, we found that the anomalous Hall sensor in this study has a high dynamic reserve $\mathrm{DRs}=20\log_{10}\left(2H_{\mathrm{DRg}}/S_{T}^{0.5}\;\left(\mathrm{at}\;1\;\mathrm{Hz}\right)\right)$ that can be calculated from the dynamic range and the field detectability. The dynamic reserve reaches 112.0 dB at 300 K and also remains at a high value of 107.7 dB at 350 K. This value is larger than both dynamic reserves of the interlayer exchange-coupled Co_40_Fe_40_B_20_ thin film-based anomalous Hall sensors (103 dB) and conventional semiconductor Hall sensors (103 dB) [2,4], and is comparable to that observed in the vortex magnetic tunnel junction-based tunneling magneto-resistance sensors (115 dB) [50]. The high dynamic reserve allows our anomalous Hall sensors to be able to measure magnetic field accurately, even under a large background magnetic field.

## 4. Conclusions

In conclusion, we have successfully fabricated an energy-efficient anomalous Hall sensor based on soft-magnetic Fe*_x_*Pt_1−*x*_ thin films with the magnetization controlled to be near the spin-reorientation transition. The magnetization was tuned by varying the Fe*_x_*Pt_1−*x*_ composition, the Fe*_x_*Pt_1−*x*_ layer thickness, its interfacial oxide layers, the post-growth annealing temperature, and the measurement temperature. The best performance was found in an as-grown multilayer of MgO(1.6)/Fe_0.48_Pt_0.52_(1.25)/MgO(1.6)/TaO*_x_*(1.0) at room temperature, characterized by a large anomalous Hall angle of 1.95%. The sensor features a sensitivity of 644.1 Ω/T at zero field, a large dynamic range of 949 Oe, and a field detectability of 237.5 nT/√Hz at 1 Hz and 15.3 nT/√Hz at 10 kHz at a zero-bias field. Notably, the anomalous Hall sensor has a high dynamic reserve of 112.0 dB, among the highest values in all the known Hall sensors and magneto-resistive (MR) sensors.

These results suggest that the Fe*_x_*Pt_1−*x*_ alloy is a suitable solid system for constructing highly energy-efficient anomalous Hall sensors, due to its strong inherent spin–orbit interaction (leading to a large Hall angle) and strong spin polarizability of Pt. We conjecture that the magnetic sensing performance can be enhanced, perhaps significantly, by fabricating Fe*_x_*Pt_1−*x*_-based interlayer exchange-coupled magnetic thin films with further improvement of the PMA.

## Data Availability

The authors declare that the data supporting the findings of this study are available within the article and are available from the corresponding author upon reasonable request.
